# Photocatalytic Antibacterial and Antibiofilm Activities of Subphthalocyanine/TiO_2_ Derivates: Integrating Experimental Findings with Molecular Docking and Dynamics

**DOI:** 10.1021/acsomega.5c01465

**Published:** 2025-05-28

**Authors:** Buket Guntay, Tugce Ozcan, Sifa Dogan, Ilknur Aksoy Çekceoğlu, Gülbin Kurtay, Emre Aslan, Mine Ince, Imren Hatay Patir

**Affiliations:** † Department of Biochemistry, 52993 Selcuk University, Konya 42130, Turkey; ‡ Department of Biotechnology, 563761 Selcuk University, Konya 42250, Turkey; § Department of Natural and Mathematical Science, Tarsus University, Mersin 33400, Turkey; ∥ Department of Chemistry, Hacettepe University, Ankara 06800, Turkey

## Abstract

The antibacterial and antibiofilm properties of SubPcs were investigated against the pathogenic bacteria E. coli, S. aureus, and MRSA. SubPcs have demonstrated the highest efficacy against S. aureus, achieving antibacterial effects of around 100% through LED light exposure. This was followed by the observed effects of 76% on E. coli and 56% on MRSA. The GSH depletion assay revealed that SubPc-3 demonstrated the highest depletion rate of 82% when exposed to LED light. Fluorescence and SEM analyses were carried out to confirm bacterial membrane damage activities of SubPcs. Molecular docking and dynamics simulations were applied for the understanding of the molecular mechanisms. The *in-silico* investigations offered useful insights into the potential binding mechanisms and interactions of SubPcs with the essential bacterial proteins. These findings supported our experimental results and gave molecular reasons for the observed antibacterial effects. Furthermore, the photocatalytic antibacterial mechanism of SubPcs/TiO_2_ has been clarified by analyzing the electronic band levels of SubPcs and TiO_2_. This analysis has provided insights into the mechanism of ROS formation under light irradiation. The combination of experimental and computational methods in this study not only shows the effectiveness of SubPcs but also enhances our comprehension of their molecular mechanisms. Hence, it is hypothesized that these harmless SubPcs will provide valuable insights for future studies on non-antibiotic photochemical antimicrobials, presenting a hopeful strategy to combat antibiotic-resistant bacteria by utilizing both photocatalytic activity and specific protein interactions.

## Graphical abstract

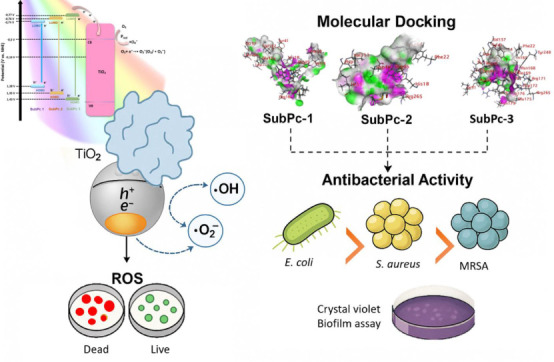

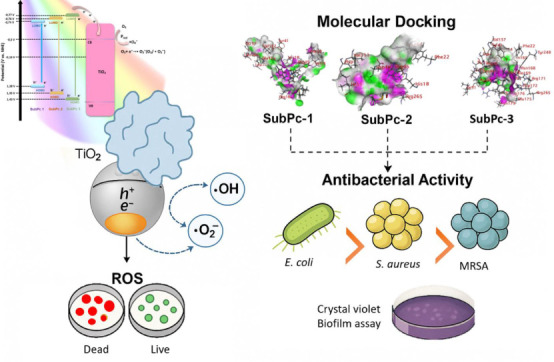

## 1 Introduction

The past few decades have witnessed a critical rise in bacterial resistance to various antibiotics, posing a significant medical challenge across healthcare systems.^1^ Over a decade ago, the Centers for Disease Control and Prevention (CDC) issued a warning that we are nearing the “post-antibiotic era,” underscoring the urgent need for innovative solutions to curb bacterial proliferation.^2^ Photocatalytic antibacterial process is based on the reaction between the reactive oxygen species (ROS) and bacterial cells. ROS, as an intermediate, outcomes from a deficient reduction reaction that exhibits advanced activity between O_2_ or water.^3^ ROS mainly contains singlet oxygen (•O_2_), superoxide anions (•O_2_^–^), hydroxyl radicals (•OH), etc. The photocatalytic antibacterial pathway includes the irradiation with light of a specific wavelength, which excites the photosensitizers (PSs) to produce photogenerated electrons or holes. These photogenerated electrons or holes can react with O_2_ or water to generate highly active radical molecules, which can attack the bacterial cell and cellular components.^4^ Gram-positive bacteria are very sensitive to the photocatalytic process-dependent ROS attack, whereas Gram-negative bacteria show remarkable resistance to negatively charged or neutral substances owing to their extra outer membrane.^5^ However, currently available materials come with certain limitations, such as poor water dispersibility and nonspecific photosensitizer residue. Efforts are also made to prevent aggregation and enhance the efficacy of nanomaterials.^6^ Nevertheless, due to their hydrophobic nature, phthalocyanines have a tendency to aggregate in aqueous solutions, which consequently restricts their applicability in photocatalytic and photodynamic biological processes. However, subphthalocyanines (SubPcs) decreasing the probability of aggregation due to their 3D chemical structures.^7^ Taking all these perspectives into consideration, advancements in nanomaterials are expected to play a crucial role in addressing the pressing antibiotic resistance issue.^8,9^ Antibiotic therapy remains a primary strategy for combating bacterial infections. However, the unconscious consumption of antibiotics have led to the rise of antibiotic-resistant bacterial strains, diminishing the effectiveness of conventional treatments.^10^ However, although nanomaterials may be unable to penetrate the cellular pores, they can still induce cell death by disrupting the structural integrity of essential intracellular proteins, resulting in nonresistant antibacterial effects.^11^

There exists a wide array of highly efficient PSs with photocatalytic antimicrobial properties, including porphyrins, chlorins, phthalocyanines, and other nanostructures.^12–14^ Phthalocyanine-based nanomaterials are known as suitable PSs for photoinactivation of pathogenic microorganisms due to their physicochemical properties. SubPcs, known as the lowest homologues of phthalocyanines (Pcs), possess 14 π-electron aromatic macrocycles including three diiminoisoindole rings around a central boron core.^15^ The mechanism of photosensitizers relies on the administration of a photosensitizing agent, which preferentially accumulates within microbial cells. Upon subsequent irradiation with visible light in the presence of oxygen, ROS are generated, leading to cellular damage and the inactivation of microorganisms. SubPcs are fluorescent materials that easily can generate •O_2_ under visible light irradiation^16^ and could be as a non-antibiotic approach for the pathogenic bacterial treatment due to the enhanced ROS production with visible light irradiation, which causes membrane or cell damage of the microorganisms and can overcome the resistance issue.^7^ Given the diminishing development of novel antibiotics, there is an urgent demand for alternative therapeutic approaches to combat multidrug-resistant (MDR) bacteria, particularly those capable of inhibiting the progression of resistance and preventing the emergence of new resistant strains. Such alternatives play a vital role in antimicrobial stewardship.^17^ In this context, light-activated photodynamic therapy (PDT) has gained significant attention as a promising strategy for bacterial infection treatment due to its noninvasive nature and favorable safety profile.^18^ Antimicrobial photodynamic therapy (aPDT) is an adaptation of PDT, fundamentally relying on the interaction of three key components: a specific wavelength of light, a photosensitizing agent, and molecular oxygen.^19^

In this context, in a study conducted in 2021, Mapukata et al. demonstrated its antibacterial effect by testing Pc@Ag-SiO_2_ nanofiber (NF) structures on Staphylococcus aureus bacteria under visible light.^9^ In another study, Biyikoglu et al. demonstrated the inhibitory effects of photodynamic therapy (PDT) on S. aureus and E. coli bacteria by using substituted silicon phthalocyanine (Es-SiPc) and subphthalocyanine (Es-SubPc) structures in 2019.^20^ Antibacterial effects of zinc phthalocyanine (ZnPc)-based poly­(vinyl alcohol)­s (PVA-Pcs) were demonstrated in 2020 using Gram-negative (Gr(−), E. coli and Salmonella*typhimurium*) and Gram-positive bacteria (Gr­(+), S. aureus and Listeria monocytogenes).^21^ Our group previously demonstrated the antibacterial effects of TiO_2_-dependent ZnPc structures against E. coli and S. aureus bacteria under light-emitting diode (LED) light.^22^ ZnPcs were also shown with their in vitro and in vivo PDT-driven antitumor effects (up to 98.7%) of HepG2 cancer cells. In the same study, ZnPc exhibited photodynamic inactivation against S. aureus.^6^ SubPc/TiO_2_ has been reported as a significant phototoxic agent against mammary and cervical tumor cell lines.^23^ SubPc was used as a suitable tumor imaging agent with PDT in colon carcinoma cells and demonstrated as a nuclear imaging agent when labeled with radionuclei in carcinoma using white LED light.^24^ SubPc boron­(III) iodide (SubOPc^3+^) was shown to inhibit E. coli under visible light with PDT and PDI.^16^ The study with SubPc-integrated TiO_2_s showed also good antibacterial effects with PDT on E. coli and S. aureus bacteria.^7^ Roy et al. demonstrated that a multifunctional SubPc polymer nanosphere has superior photo-inactivation ability on E. coli.^25^ All these studies have shown that SubPcs have PDT, PDI, and/or photocatalytic antitumor and antimicrobial effects under exposure of visible light irradiation.

Herein, SubPc-1, SubPc-2 and SubPc-3 having carboxylic acid as anchoring group were immobilized on TiO_2_ nanoparticles. SubPc-1, SubPc-2 and SubPc-3 features a bisthiophene, propyl-thioether functional groups, and nonfunctional group, respectively. The antibacterial and antibiofilm properties of SubPcs/TiO_2_ were examined on E. coli, S. aureus and methicillin-resistant S. aureus (MRSA) under white LED light. Notably, this study has pointed out the first utilization of SubPcs sensitized TiO_2_ in an antibacterial study. Bacterial viability was assessed under white LED light through hourly monitoring of optical density (OD_600nm_). The abilities of ROS generation by SubPc/TiO_2_ derivates have been investigated via a glutathione (GSH) oxidation assay. Fluorescent microscopy analysis was performed by Live–Dead staining kit to assess membrane damage. In addition, morphological defects in bacteria were visualized using scanning electron microscopy (SEM). Furthermore, the crystal violet biofilm assay was performed to assess the biofilm efficacy of the synthesized SubPc/TiO_2_, confirming their antibiofilm activity. To further elucidate the potential mechanisms of action and binding interactions of the target SubPc derivatives, molecular docking and dynamics studies were performed using specific protein targets (CrtM, DNA gyrase, and Penicillin-binding protein 2a) for each bacterial strain. On this basis, SubPc-1 and SubPc-2 generally exhibited stronger binding affinities compared to SubPc-3 across all targets, aligning with experimental antibacterial results. To further investigate the stability and dynamic behavior of the SubPc derivatives in complex with their target proteins, we conducted extensive molecular dynamics (MD) simulations over a 100 ns timeframe. According to Root Mean Square Deviation (RMSD) analyses, SubPc-2 demonstrated remarkable stability in complex with PBP2a, while SubPc-1 maintained consistent interactions with CrtM, corroborating their strong docking scores. Solvent Accessible Surface Area (SASA) and Radius of Gyration (Rg) trajectories further elucidated the nature of these interactions, indicating that while the SubPc derivatives may induce subtle conformational changes in their target proteins, they do not disrupt the overall structural integrity. The combination of strong binding affinities and stable complex formation over time suggests that these compounds may exert their effects through both specific protein interactions and their photocatalytic properties, highlighting their potential as multi-modal antibacterial agents.

## 2 Experimental Section

### 2.1 Materials

E. coli (ATCC 25922), MRSA (ATCC 43300), and S. aureus (ATCC 25923) were provided from the microorganism culture collection of Vocational School of Health Services (Selcuk University). The SubPcs/TiO_2_ derivatives were acquired from our preceding study and were characterized in detailed examination by means of structural and morphological techniques in our previous study.^26^ The molecular structures of SubPcs are given in Figure 1. Additionally, scanning electron microscopy (SEM) was also used to study the morphologies of SubPc-1/TiO_2_, SubPc-2/TiO_2_ and SubPc-3/TiO_2_. As can be seen in Figure S1, there is no significant morphological differences among these materials. Phosphate-buffered saline (PBS), reduced l-glutathione (GSH), 5,50-dithiobis­(2-nitrobenzoic acid (DNTB), and glutaraldehyde were purchased from Sigma-Aldrich. Crystal violet (CV), glacial acetic acid, tryptic soy broth (TSB) medium, and ethanol for spectroscopy were purchased from Merck.

**Figure 1:**
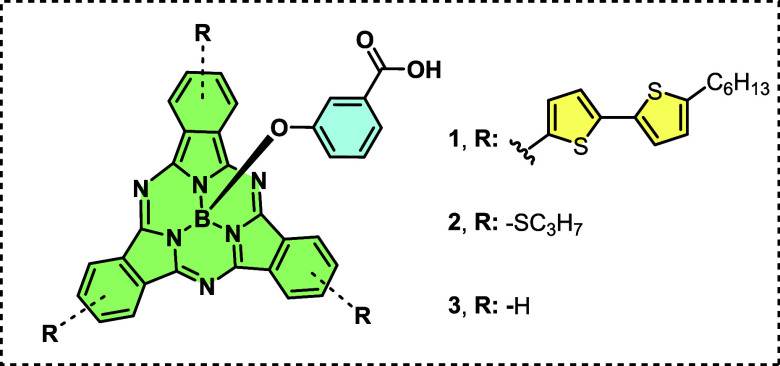
Organic representation of SubPc structures.

### 2.2 Antibacterial Analysis

The SubPc-1-2-3/TiO_2_ powder samples were prepared in pure water at a concentration of 64 μg/mL and then sonicated for 20 seconds to ensure the dispersion of the catalysts. Antibacterial experiments have been conducted using white LED light illumination (λ > 400 nm, 5 W·m^–2^).^27^ E. coli, MRSA, and S. aureus strains were pre-cultured in TSB broth at 37°C for 12 h. The final concentrations of bacterial cultures were adjusted to 2 × 10 ^6^ CFU mL^–1^ by diluting them in TSB broth, and later, they were added to a 96-well plate. The experiments were divided into six sections, each of which was replicated as follows: (1) bacteria (E. coli, S. aureus, or MRSA), (2) SubPc-1/TiO_2_, (3) SubPc-2/TiO_2_, (4) SubPc-3/TiO_2_, (5) SubPc-1/TiO_2_/LED, (6) SubPc-2/TiO_2_/LED, (7) SubPc-3/TiO_2_/LED. LED light was applied for 30 min to groups 4, 5, and 6. Afterward, the entire plates were incubated at 37°C for 12 h. The antibacterial activities of SubPcs against E. coli, MRSA, and S. aureus were tested using a microplate reader with OD_600 nm_ measurements.

### 2.3 GSH Consumption Assay

The GSH depletion experiment was conducted both in the presence of LED light and in a dark environment to demonstrate the presence of ROS species in the environment. Since the decrease in GSH levels is directly proportional to the amount of ROS generated in the experimental environment, it serves as a supporting factor in explaining the antibacterial activity mechanism. The experiment is based on measuring 5-thio-2-nitrobenzoic acid (TNB) at OD_412 nm_, resulting from the reaction between dithiobis­(2-nitrobenzoic acid) (DNTB) and GSH. The experimental groups were arranged as follows: (1) GSH, (2) H_2_O_2_, (3) SubPc-1/2/3, and (4) SubPc-1/2/3/LED. Group 4 was subjected to 30 min of LED irradiation. GSH in PBS was added to each group, and 2 mM H_2_O_2_ (used as a positive control) was employed to initiate the oxidation of GSH. After 4 h of incubation in the dark, 90 mL of each sample was mixed with 10 mL of Ellman reagent solution in PBS and DMSO (2 mM).^28^ Following a 15 min incubation, OD_412 nm_ was measured using a microplate reader.

### 2.4 Morphologic Analysis of Bacteria with SEM

The impact of SubPc-1/TiO_2_, SubPc-2/TiO_2_, and SubPc-3/TiO_2_ structures on the structural integrity of bacteria was assessed by using scanning electron microscopy (SEM). Bacteria at the desired concentration were incubated with these compounds at 37°C for 3 h and exposed to LED light for 30 min. At the end of the third hour, following two washes with 0.1 M PBS (pH 7.2), each sample was treated with glutaraldehyde solution for fixation and incubated for an additional 2 h. Dehydration was performed twice at different concentrations (50% and 100%) of ethanol for 10 min each time. Subsequently, the samples were left to air-dry for 1 day at a temperature of 25°C before undergoing microscopic analysis.

### 2.5 Biofilm Test with Crystal Violet

The crystal violet assay was employed to investigate the influence of nanomaterials on bacterial biofilm formation. Initially, bacteria were incubated overnight in a microplate. Subsequently, the wells were washed three times with 200 μL of PBS to eliminate planktonic bacteria and then allowed to air-dry. For staining the biofilm layer, 200 μL of crystal violet was added to the dried wells and incubated for 15 min. Each well was washed twice with 100 μL of distilled water to remove any excess dye not bound to the biofilm matrix. Finally, 200 μL of 33% acetic acid was added, and the absorbance was measured at 570 nm using a Thermo Scientific Multiskan GO microplate reader. The antibiofilm activities (%) of SubPc-1/TiO_2_, SubPc-2/TiO_2_, and SubPc-3/TiO_2_ were calculated using the equation provided below (eq 1).

1 Antibiofilm effect(%)=((C−B)−(T−B)/(C−B)×100

B represents the average absorbance value per well for the negative control (medium only), C represents the average absorbance value per well for the positive control (no treatment), and T represents the average absorbance value per well for compound-treated wells.

### 2.6 Molecular Docking

The molecular structures of SubPc-1, SubPc-2, and SubPc-3 were initially constructed using GaussView 6.0 software. To ensure accurate representation of the compounds’ conformations in aqueous environments, we performed quantum mechanical calculations using Gaussian 09. Geometry optimizations and frequency calculations were carried out using Density Functional Theory (DFT) with the B3LYP hybrid functional and the 6-31G­(d,p) basis set. These calculations employed the SMD (Solvation Model based on Density) implicit solvent model, simulating the compounds in water. Following optimization and frequency analysis, the structures were confirmed to be at true energy minima by the absence of imaginary frequencies. The optimized geometries were then converted to .mol2 format. DFT-optimized SubPc molecules were used as inputs for ligand molecules, and corresponding docking simulations were carried out using the AutoDock Vina extension implemented in the SAMSON platform (2024-R1, OneAngstrom). The X-ray crystal structures of CrtM (PDB ID: 3ACW), DNA gyrase (PDB ID: 4DUH), and PBP2a (PDB ID: 1MWT) were obtained from the Protein Data Bank (www.rcsb.org) (Figure 2a–c). Water molecules were removed from all macromolecules, and charges and hydrogens were added to the structures of CrtM, DNA gyrase, and PBP2a for docking. For PBP2a (1MWT), Chain A was deleted, and Chain B was chosen for the molecular docking procedure. For DNA gyrase (4DUH), Chain B was deleted, and Chain A was selected for analysis. The CrtM (3ACW) structure, containing only Chain A, was used in its entirety. The docking simulations were performed using a grid box centered on the active site or known binding pocket of each protein. Multiple poses were generated for each SubPc derivative and ranked based on their predicted binding affinities. Corresponding analysis and visualization of the SubPc-protein complexes were performed using both SAMSON and Discovery Studio Visualizer 2024 (client version; Accelrys Software Inc., San Diego, CA, USA).

**Figure 2:**
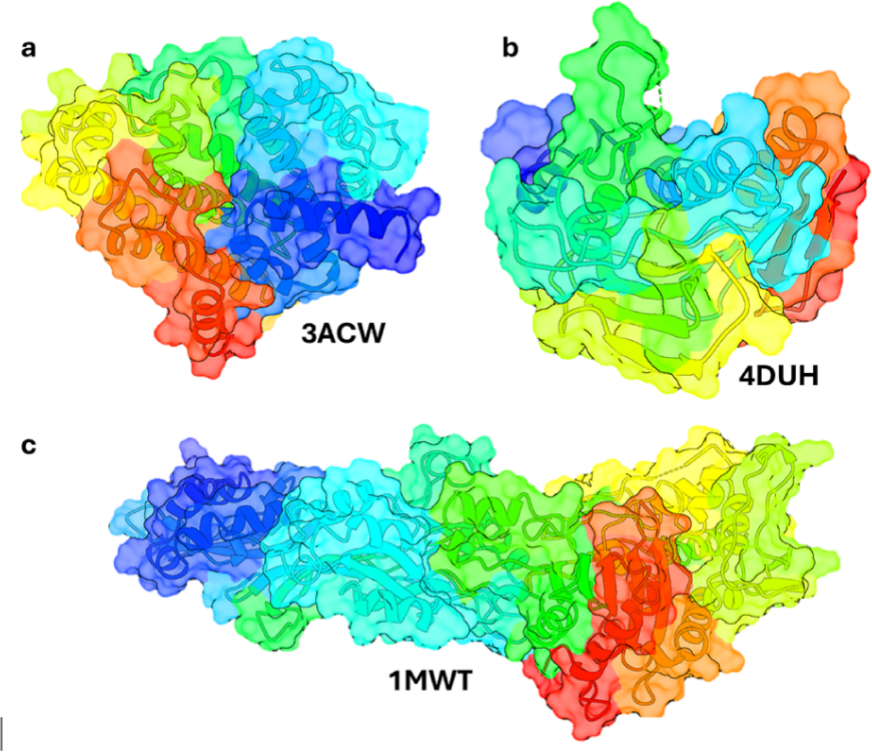
(a) CrtM protein (PDB ID: 3ACW) to represent the S. aureus. (b) DNA gyrase enzymes (class topoisomerase II) (PDB ID:4DUH) for E. coli. (c) Penicillin G acyl-penicillin binding protein 2a (PDB ID: 1MWT) from MRSA.

### 2.7 Molecular Dynamics

Molecular dynamics (MD) simulations were conducted using the GROningen Machine for Chemical Simulations (GROMACS) 2023.3 software package.^29^ The CHARMM36 force field and TIP3P water model were selected for the preprocessing step. Electrostatic interactions were computed using the Particle Mesh Ewald (PME) method, with a Fourier spacing of 0.16 nm and a short-range cutoff of 1.2 nm. System neutralization was achieved by adding chloride ions, followed by energy minimization using the steepest descent method for 5000 steps or until the maximum force was less than 10.0 kJmol^–1^nm^–1^. System equilibration was performed in two phases: a constant number of particles, volume, and temperature (NVT) ensemble, and a constant number of particles, pressure, and temperature (NPT) ensemble. The temperature was gradually increased to 300 K over 100 ps in the NVT ensemble using the Berendsen Thermostat, followed by 100 ps in the NPT ensemble at 1 bar. Production MD simulations were run for 100 ns for all complexes. Various analyses were performed using the GROMACS package, including root-mean-square deviation (RMSD), solvent-accessible surface area (SASA), and radius of gyration (Rg). These calculations provided insights into the dynamic behaviors of the investigated systems, allowing for a comprehensive evaluation of the stability and interactions of the SubPc derivatives with their target proteins over time.

## 3 Results and Discussion

### 3.1 Antibacterial Activity

Antibacterial activities of SubPc-1/TiO_2_, SubPc-2/TiO_2_, and SubPc-3/TiO_2_ were investigated against E. coli, S. aureus, and MRSA by optical density measurement at 600 nm. After the application, the bacterial growth curve graph of the dark and light conditions was created (Figure 3). Antibacterial growth curves for 4 hours have been shown in Figure 3b,d,f. As indicated in the bar graphs, the antibacterial percentages for E. coli (Figure 3a) were found to be SubPc-1/TiO_2_ as 40%, SubPc-2/TiO_2_ as 27%, and SubPc-3/TiO_2_ as 17% in the dark field. For the same bacteria, the antibacterial effects were increased to be 76%, 71%, and 69% for SubPc-1/TiO_2_, SubPc-2/TiO_2_, and SubPc-3/TiO_2_, respectively, under LED light. As indicated in the antibacterial bar graphs for S. aureus (Figure 3c), their activities were calculated as 36%, 46%, and 35% for SubPc-1/TiO_2_, SubPc-2/TiO_2_, and SubPc-3/TiO_2_ in the dark conditions, respectively. However, in LED light conditions, the antibacterial activities were increased to nearly 100% for SubPc-1/TiO_2_ and SubPc-2/TiO_2_ and to 96% for SubPc-3/TiO_2_. The percentage of antibacterial effect in MRSA, which is seen in the most resistant bacterium among them, has shown as 23%, 25%, and 15% for SubPc-1/TiO_2_, SubPc-2/TiO_2_, and SubPc-3/TiO_2_, respectively, in the dark medium. Under LED light irradiation, the antibacterial effects were calculated as 31%, 56%, and 20% for SubPc-1/TiO_2_, SubPc-2/TiO_2_, and SubPc-3/TiO_2_, respectively. As seen from the antibacterial activity studies, SubPc/TiO_2_ structures were most effective on S. aureus bacteria, which was found in accordance with other SubPcs studies in the literature.^16^ Consistent with all these results, SubPc/TiO_2_ samples demonstrated enhanced antibacterial efficiency under LED light irradiation because the material was stimulated under LED light irradiation to produce more ROS, which is stated in the mechanism part in detail. E. coli is a Gram-negative bacterium with an outer membrane composed of lipopolysaccharides. This structure provides an additional protective barrier, which restricts the penetration of reactive oxygen species (ROS) generated during photodynamic treatment. As a result, E. coli is more likely to survive the initial oxidative stress and adapt during the lag phase without immediate cell death^30^ Moreover, E. coli exhibits a robust oxidative stress response, involving key enzymes such as catalase and superoxide dismutase, which effectively neutralize ROS and facilitate cellular recovery and delayed growth.^31^ Furthermore, studies in the literature have highlighted that E. coli possesses a self-repair capability attributed to its outer membrane structure. This structural feature may partially reverse the effects of antibacterial agents, leading to a slower cell death rate compared to Gram-positive bacteria.^32^

**Figure 3:**
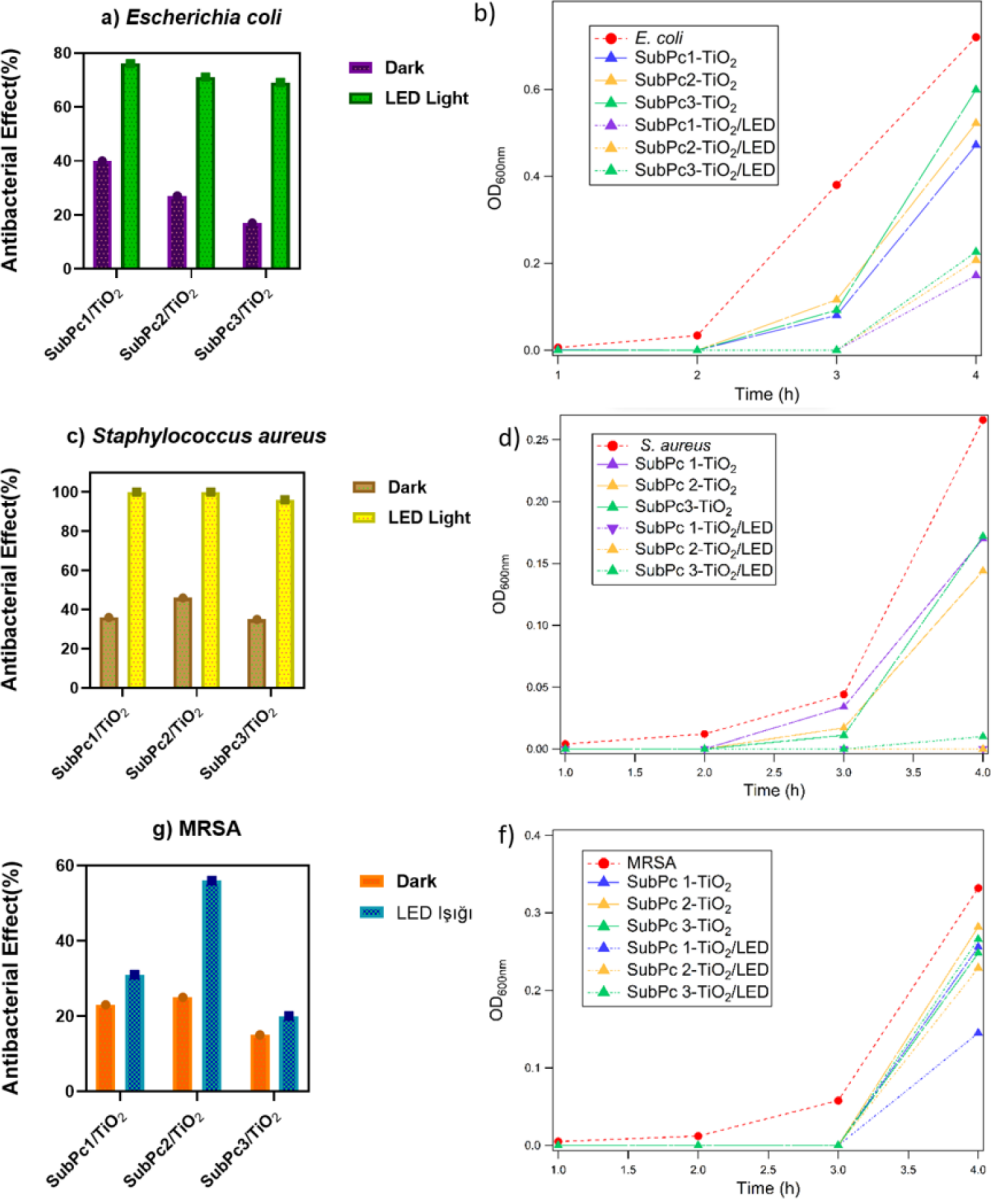
4-hour antibacterial growth curve and antibacterial effect percentage bar graphs; E. coli (a), S. aureus (c), and MRSA (e) antibacterial effect percentage graphs; E. coli (b), S. aureus (d), and MRSA (f) antibacterial growth curve.

The antibacterial wet lab results are further supported by the integration of molecular docking findings. This approach provides a more comprehensive explanation of the molecular mechanisms behind the interactions between SubPc derivatives and bacterial proteins, reinforcing the observed antibacterial efficacy.

### 3.2 Photocatalytic GSH Activity

GSH consumption of SubPcs/TiO_2_ were measured to elucidate the antibacterial mechanism resulting from ROS-dependent photocatalytic antibacterial activities. The GSH consumption percentages were found as 25.5%, 33% and 40.5% for SubPc-1/TiO_2_, SubPc-2/TiO_2_ and SubPc-3/TiO_2_, respectively, in the dark conditions (Figure 4a). Then, the amount of GSH depletions were found to be 59%, 75.5% and 83% for SubPc-1/TiO_2_, SubPc-2/TiO_2_ and SubPc-3/TiO_2_, respectively, in the presence of LED light irradiation (Figure 4b). The amount of GSH consumed appears to be higher in light compared to the antibacterial effect percentages that occur under LED light irradiation, which is explained by ROS formation causing damage the cell membrane.

**Figure 4:**
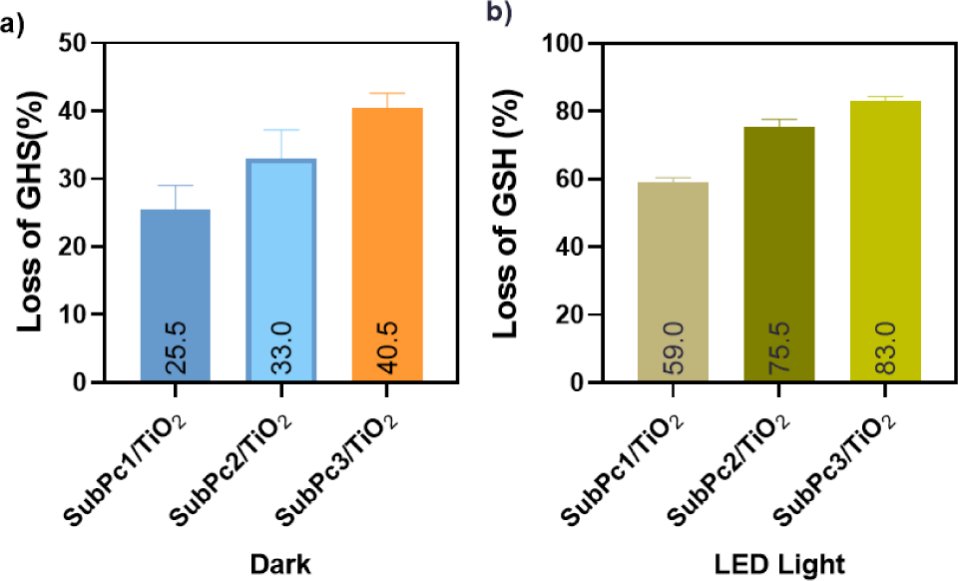
GSH depletion results: (a) in dark conditions and (b) under LED light irradiation.

### 3.3 Antibiofilm Activity

Biofilm inhibition effects of SubPcs/TiO_2_ were tested by crystal violet colorimetric analysis as shown in Figure 5. The results showed that SubPcs/TiO_2_ worked more effectively under LED light irradiation. Biofilm inhibition OD values of E. coli bacteria (Figure 5b) are given, and Figure 5a shows that antibiofilm activities of SubPc-1/TiO_2_, SubPc-2/TiO_2_, and SubPc-3/TiO_2_ on E. coli were found to be 72%, 70% and 76% in the dark, respectively. The antibiofilm effects on E. coli under LED light were shown to be 83%, 89% and 95% for SubPc-1/TiO_2_, SubPc-2/TiO_2_ and SubPc-3/TiO_2_, respectively. Biofilm inhibition OD values are given for S. aureus (Figure 5c,d), while percentages of biofilm inhibition were found to be 36%, 46% and 35% in the dark conditions, while they were stated as 79%, 73% and 83% under LED light irradiation for SubPc-1/TiO_2_, SubPc-2/TiO_2_ and SubPc/TiO_2_, respectively. These values were measured as 23%, 25% and 15% for MRSA in the dark conditions (Figure 5e,f), and 96%, 89% and 56% under LED light in the presence of SubPc-1/TiO_2_, SubPc-2/TiO_2_ and SubPc/TiO_2_, respectively. Herein, the biofilm inhibition effects of SubPc/TiO_2_ samples display in harmony with the antibacterial results mentioned above. To understand the photocatalytic antimicrobial activities, a mechanism was established regarding the redox potentials of ROS species and the band levels of SubPc/TiO_2_ derivates to explain ROS formation. As a result, the •O_2_® radical can form on the surfaces of photocatalysts produced by providing appropriate band gaps and levels. The mechanism in the absence and presence of light irradiation can be explained in mechanism part, which ROS inhibits biofilm formation through LED light irradiation and directly inhibits biofilm formation in the dark field.

**Figure 5:**
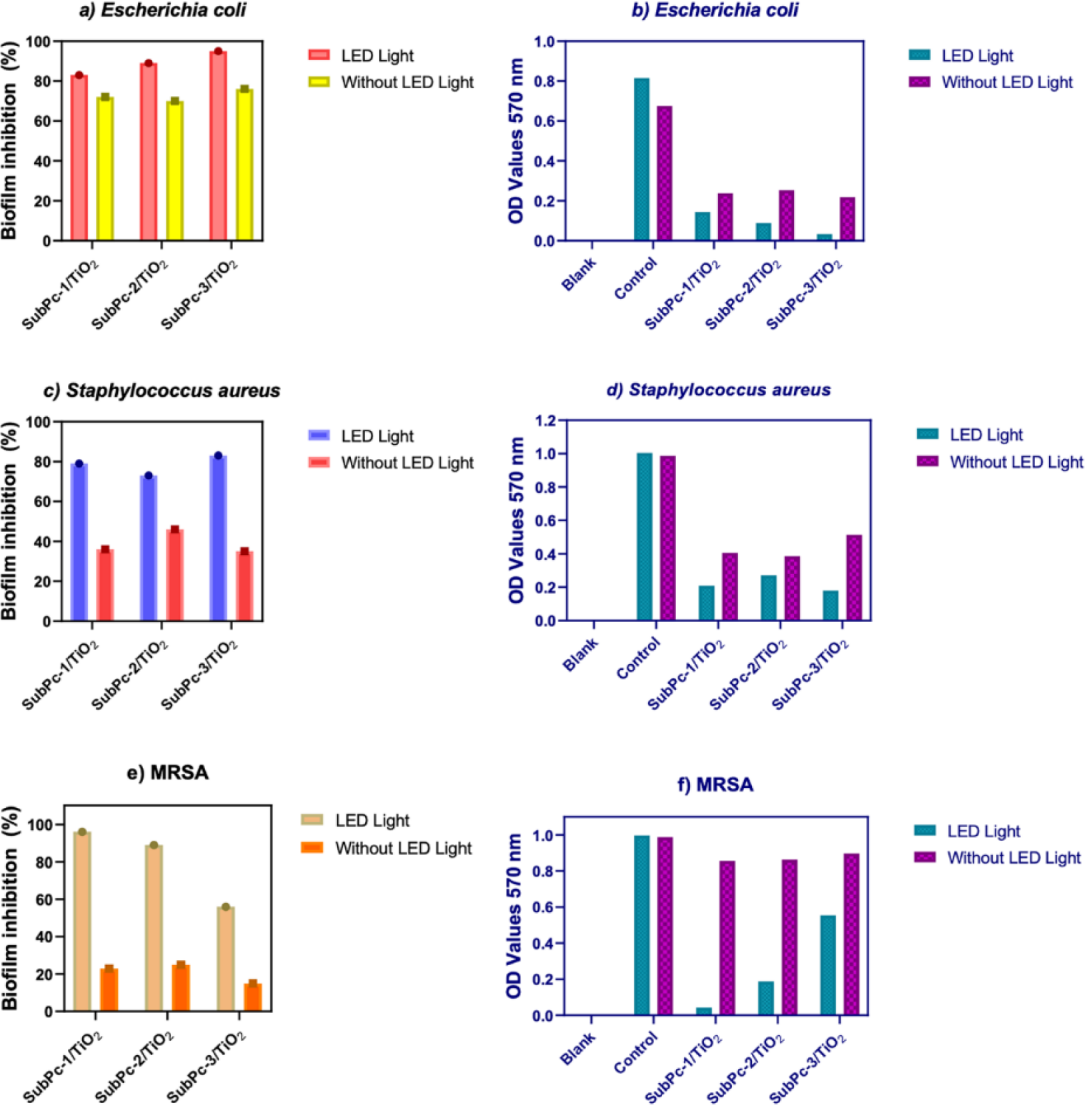
Biofilm inhibition percentages and OD_570 nm_ absorbance graphs of E. coli, S. aureus, and MRSA bacteria treated with SubPcs/TiO_2_.

The antibiofilm activity is substantiated by detailed molecular docking analyses. The computational data provide crucial insights into the molecular interactions of SubPc derivatives with key bacterial proteins, elucidating the mechanisms behind the observed antibacterial and antibiofilm effects. For instance, the strong affinity of SubPc-1 for CrtM in S. aureus (docking score: −8.592) may explain its potent antibiofilm activity. Similarly, the binding of SubPc-2 to PBP2a from MRSA (docking score: −7.032) correlates with its enhanced efficacy against biofilm formation in resistant strains, thereby reinforcing the experimental findings with molecular-level evidence

### 3.4 Fluorescence Microscopy Analysis

The bacteria treated with SubPc/TiO_2_ samples were examined using fluorescence microscopy with the Live–Dead staining kit (Figure S2). SYTO-9 binds to bacterial DNA in fluorescent images, emitting a green fluorescence when bound to intact bacterial DNA, while it emits a red fluorescence when bound to damaged or dead bacterial DNA in the presence of propidium iodide. This technique allows about the integrity of bacterial membranes (preserved (alive) and damaged (dead)). As observed in the control groups of S. aureus, MRSA, and E. coli, the SYTO-9 dye binds to undamaged DNA in live cells, resulting in green emission. Due to its lowest antibacterial effect, SubPc-3/TiO_2_ has preserved cell membrane integrity more effectively than that of SubPc-1/TiO_2_ and SubPc-2/TiO_2_ samples, emitting more green fluorescence under both dark and LED light conditions. The strongest antibacterial effect was observed in S. aureus SubPc-1–2/TiO_2_/LED, leading to the strongest red emission in the fluorescent image of S. aureus bacteria. This indicates that under LED light, the bacterial cell membranes are disrupted by the excitation of SubPc/TiO_2_ samples. Thus, the obtained antibacterial activation results above have been supported by fluorescent microscopy images accordingly.

### 3.5 SEM Analysis

SEM analysis has been employed to confirm the antibacterial activities of SubPc-1/TiO_2_, SubPc-2/TiO_2_, and SubPc-3/TiO_2_ in the absence and presence of white LED illumination by changing the morphological structures of bacterial cells and their membrane integrities. In Figure 6, the control groups of E. coli, S. aureus, and MRSA exhibited structurally typical and smooth surfaces. It was observed that the cilia and structures of all three bacteria were intact, and there was no deformation on the cell surface. In the dark, morphological changes were observed on the surface of the E. coli bacterium after treatment with SubPc-1/TiO_2_ and SubPc-2/TiO_2_, indicated by orange arrows, along with cell shrinkage due to the leakage of intracellular fluid. The antibacterial effect of SubPc-2/TiO_2_ was observed to be the highest in the dark conditions, resulting in the most cell damage in the MRSA bacterium (as indicated by violet arrows). In Figure 7, under LED light illumination, morphological damage in E. coli was characterized by cell elongation and wrinkling. However, under LED light illumination, the highest antibacterial activity was measured for SubPc-1/TiO_2_/LED and SubPc-2/TiO_2_/LED in the case of S. aureus bacterium. Consequently, deformation resembling erythrocytes (as shown by green arrows) was observed in the peptidoglycan layer of the Gr­(+) S. aureus bacterium exposed to LED light. These morphological changes suggest that SubPc/TiO_2_ samples may be originated from damaged membrane proteins and the peptidoglycan layer of bacteria. Consistent with the literature, the most significant cell surface deformation was observed on S. aureus.^22^ In line with the study conducted in 2023, molecular docking experiments were performed to measure the binding affinities of different phthalocyanines, which is derivate of SubPc to proteins on S. aureus, and higher results were obtained compared to E. coli. This is thought to be due to SubPcs binding to vital proteins in the cell wall of S. aureus.^22^

**Figure 6:**
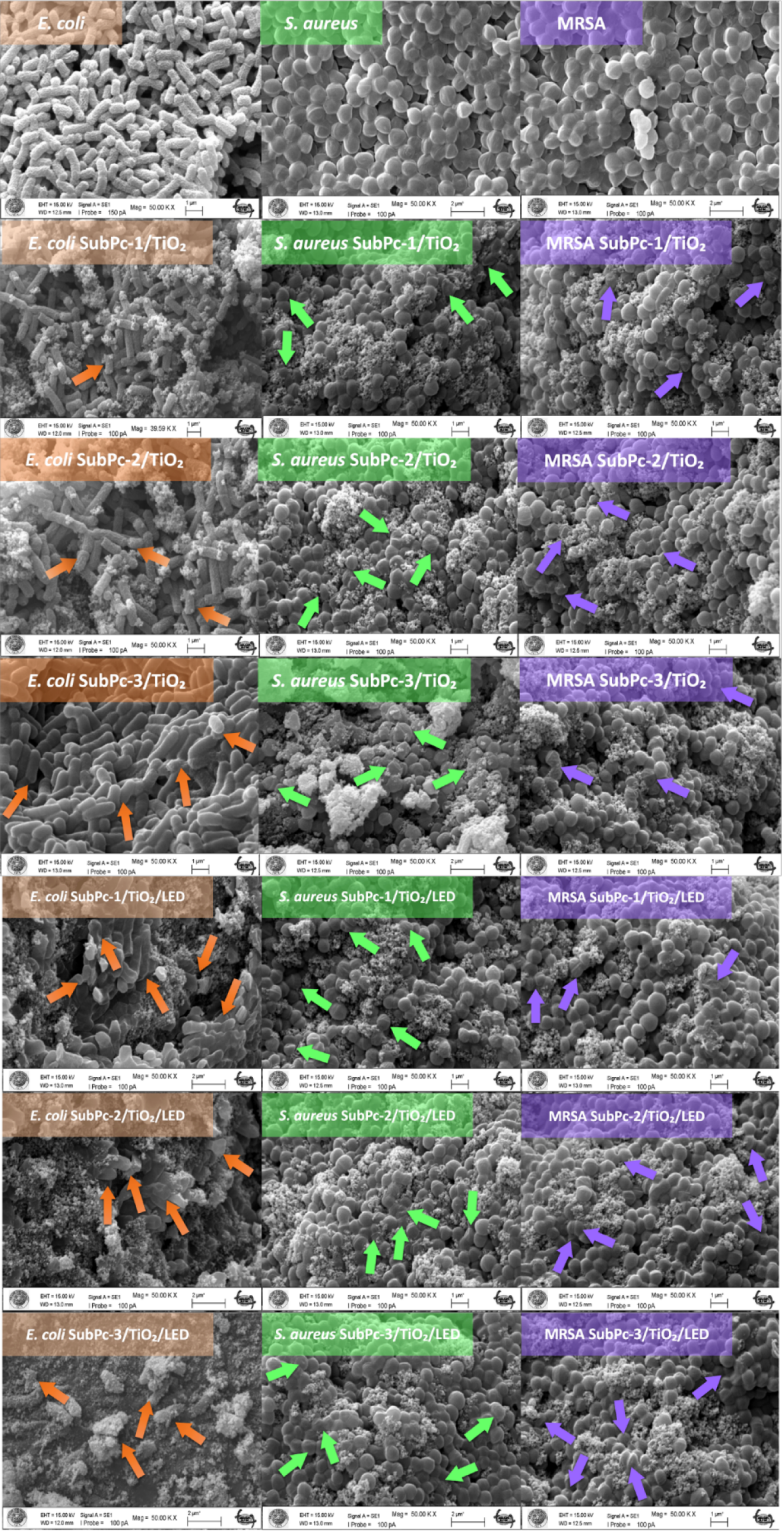
SEM images of E. coli, S. aureus, and MRSA with/without SubPcs/TiO_2_ in the absence of LED light. Scale bars are 1–2 μm. Magnification scale approximately between 40,000 and 50,000×.

**Figure 7:**
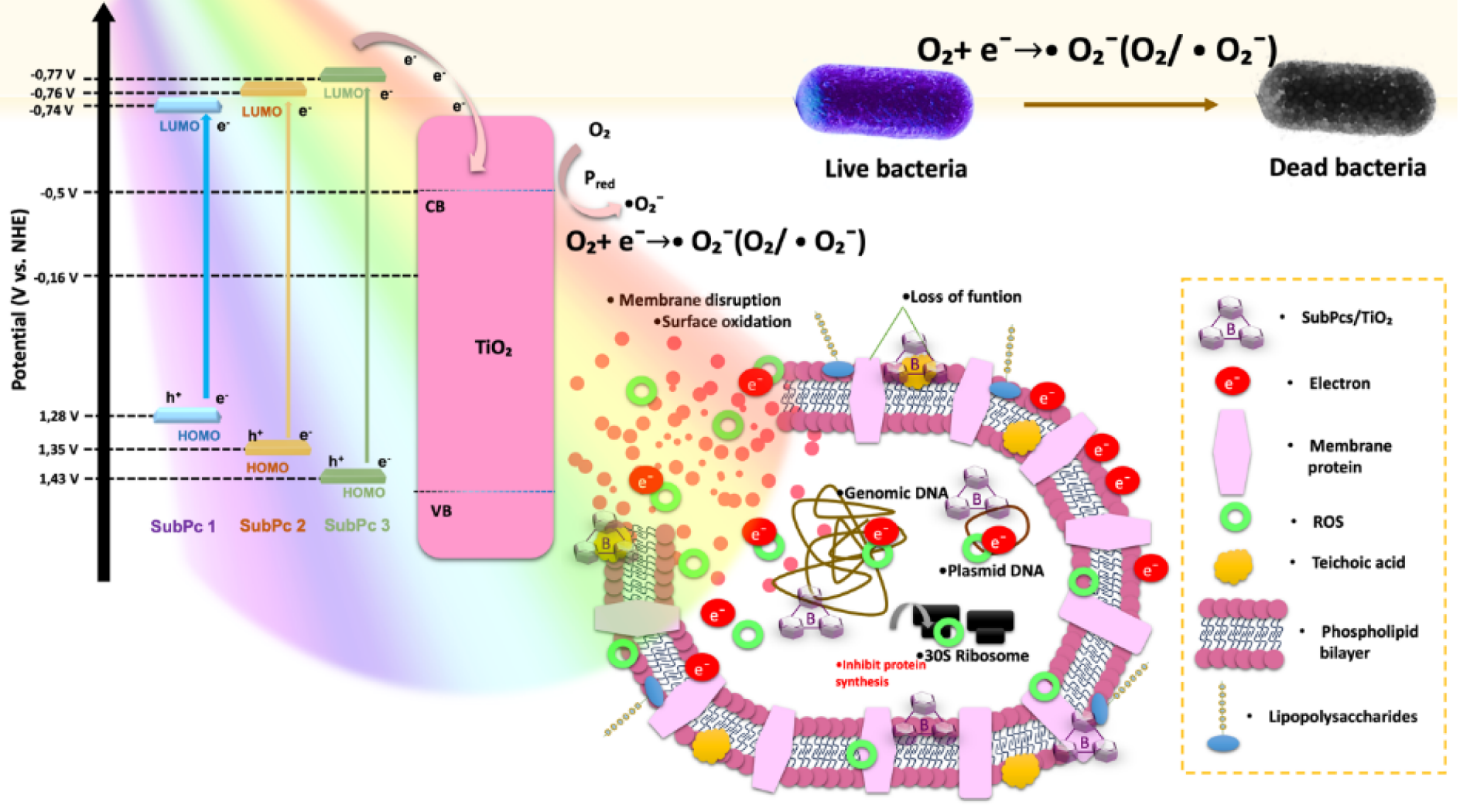
Schematic of the antibacterial mechanism occurring under possible darkness and LED light.

### 3.6 Antibacterial Mechanism

Figure 7 illustrates the photocatalytic antibacterial mechanism of SubPc-sensitized TiO_2_ under white LED light, as described by the HOMO and LUMO energy levels, which were calculated in our previous study.^26^ Firstly, SubPc molecules are stimulated by white LED light and electrons in the HOMO levels are excited to the LUMO levels. Then, the excited electrons on the LUMO levels of SubPc molecules are transferred to the conduction band (CB) level of TiO_2_, which has more positive potential than those of LUMO levels of SubPc molecules. The CB level of TiO_2_ (−0.5 eV) is more negative than the redox potential of O_2_/•O_2_^–^ (−0.33 eV), allowing photogenerated electrons in the CB of TiO_2_ to convert soluble molecular oxygen (O_2_) to the superoxide anion radicals (•O_2_^–^).^33^ These •O_2_^–^ radicals can induce oxidative stress in bacterial cells by disrupting the peptidoglycan layer, cell membrane, and intracellular components such as proteins, DNA, and RNA.^34^ In addition, the occurred •O_2_^–^ radicals can further convert to H_2_O_2_ and it is splitted and formed •OH radical, which both are act as ROS species to display antibacterial activity.^35^ In addition, TiO_2_ has wide band gap and not excited by using LED light, which is not provided ROS based antibacterial activity. Herein, the sensitized SubPc molecules enhanced the visible light absorption ability and increased dramatically antibacterial activity, which is also reported our previous phthalocyanine sensitized TiO_2_ studies for photoantimicrobial activity.^8^ Connecting the functional group moieties to the peripheral positions of SubPc-1 and SubPc-2 caused an approximately 30 nm redshift in the Q band relative to SubPc-3, which shift resulted in an increase in the photoactivity. Therefore, the enhanced photoactivity of SubPc-1 and SubPc-2 is related to the optical properties and dipole moment and led to an increase in light harvesting and charge separation efficiency, creating differences in the energy levels.^35^ The CB energy level of TiO_2_ in the SubPc/TiO_2_ structures contributed to the production of reactive oxygen species (ROS), enabling antibacterial and antibiofilm activities, which consist of the •O_2_^–^ radical.

The antibacterial mechanism was elucidated by integrating wet lab results with molecular docking analyses, focusing on key bacterial proteins. SubPc-1 exhibited a high affinity for CrtM in S. aureus (docking score: −8.592), suggesting its role in disrupting staphyloxanthin synthesis, which is crucial for bacterial virulence and biofilm formation. SubPc-2, on the other hand, demonstrated strong binding to PBP2a from MRSA (docking score: −7.032), inhibiting its role in cell wall biosynthesis, which is vital for antibiotic resistance. These protein-ligand interactions align with the observed antibacterial and antibiofilm efficacy, indicating a dual mechanism involving both ROS production and direct protein targeting.

### 3.7 Molecular Docking

To elucidate the potential binding modes and interactions of the SubPc derivatives with key bacterial proteins, we conducted molecular docking studies. The selection of specific protein chains was guided by structural and functional considerations for each target. For Penicillin-binding protein 2a (PBP2a, PDB ID: 1MWT, resolution 2.45 Å), we isolated Chain B for analysis. This choice was based on previous structural studies indicating that Chain B contains the catalytic domain and the allosteric site crucial for β-lactam resistance in MRSA. Chain B’s 646 residues encompass the key functional regions necessary for our investigation into potential inhibitor binding. In the case of DNA gyrase (PDB ID: 4DUH, resolution 2.50 Å) from E. coli, our investigation centered on Chain A. This decision was based on the structure and function of the DNA gyrase complex. DNA gyrase is a type II topoisomerase composed of two subunits: GyrA and GyrB. Chain A in the 4DUH structure represents the GyrA subunit, which is responsible for DNA binding and cleavage. This subunit contains the active site tyrosine residue that forms a covalent bond with DNA during the strand-passage reaction. The GyrA subunit is a primary target for many antibacterial compounds, including fluoroquinolones, which interfere with DNA replication by stabilizing the DNA-enzyme complex. By focusing on Chain A, we aimed to explore potential interactions between our SubPc derivatives and this therapeutically relevant site, which could provide insights into their mechanism of action against E. coli. The dehydrosqualene synthase (CrtM, PDB ID: 3ACW, resolution 1.92 Å) structure comprises only Chain A, which we utilized in its entirety. This single-chain structure represents the functional unit of CrtM, an essential enzyme in the staphyloxanthin biosynthesis pathway of S. aureus. The molecular docking studies of SubPc derivatives with PBP2a (PDB ID: 1MWT) from MRSA (Table 1) revealed diverse binding interactions and affinities. SubPc-2, featuring a propyl-thioether functional group, exhibited the highest binding affinity with a docking score of −7.032, followed by SubPc-1 (−5.801) and SubPc-3 (−5.013). SubPc-2, despite its higher binding score, showed fewer specific interactions. The main interactions observed were hydrophobic, including π–π T-shaped interactions with TYR446 and HIS583, and an alkyl interaction with VAL448. SubPc-1, containing a bisthiophene functional group, showed intermediate binding affinity. The extended π-system of the bisthiophene moiety appears to facilitate multiple π-π stacking interactions, particularly with TYR446. This conjugated system may contribute to the compound’s ability to form a network of non-covalent interactions within the protein binding site. The compound also engaged in various hydrophobic interactions, including π-alkyl interactions with ILE512 and TYR441. On the other hand, SubPc-3 displayed a different interaction profile, with conventional hydrogen bonds formed with LYS430 and ASN464. Carbon hydrogen bonds were also observed with several residues including LYS430, TYR446, and THR444. Hydrophobic interactions included π-sigma with TYR446 and π-alkyl with LYS430.

**Table 1:**
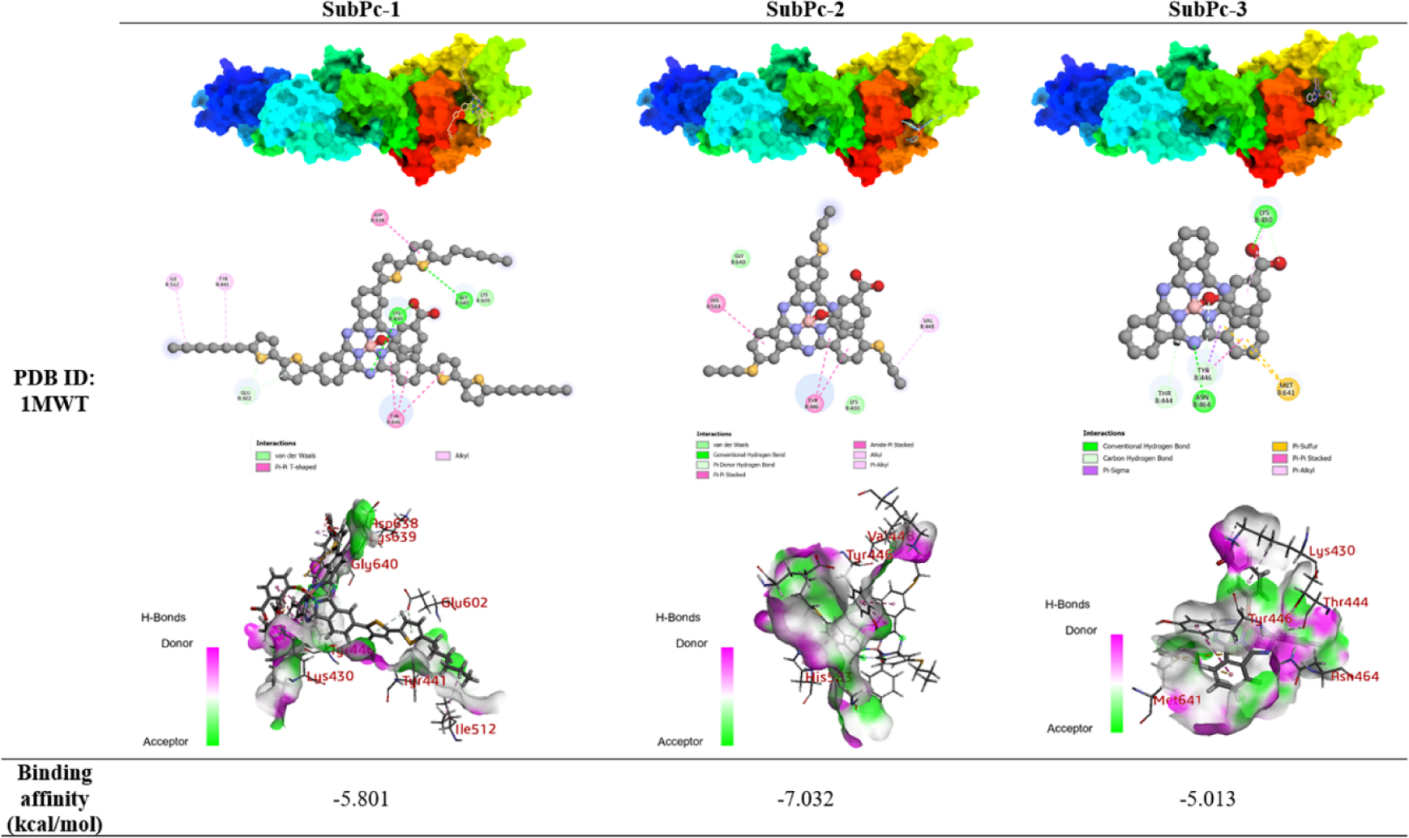
Binding Poses and Residue Interactions of the SubPcs with Penicillin-Binding Protein 2a (Pbp2a, PDB ID: 1MWT) from MRSA

Similarly, the molecular docking studies of SubPc derivatives with DNA gyrase (PDB ID: 4DUH) from E. coli revealed interesting insights into their binding affinities and interaction patterns (Table 2). SubPc-2 exhibited the highest binding affinity with a docking score of −6.311, closely followed by SubPc-1 (−6.282). SubPc-3 showed considerably lower affinity (−3.998). SubPc-2’s superior binding can be attributed to a balanced combination of hydrogen bonding and hydrophobic interactions. It formed a conventional hydrogen bond with ARG76 and engaged in multiple hydrophobic interactions, predominantly alkyl and π-alkyl, with residues such as ALA90, VAL93, ILE94, and PRO79. The flexibility of the propyl-thioether group likely allows for optimal positioning within the binding pocket, maximizing favorable contacts. SubPc-1, despite its slightly lower binding score, demonstrated a more diverse interaction profile. It formed a conventional hydrogen bond with GLU137 and participated in numerous hydrophobic interactions, including a π-π T-shaped interaction with HIS55 and various alkyl and π-alkyl interactions with residues like LYS57, ILE78, ILE94, and ARG76. The extended π-system of the bisthiophene group appears to facilitate these additional aromatic interactions, contributing to its high affinity. SubPc-3, while showing the lowest affinity, still formed two conventional hydrogen bonds with ARG136. However, its hydrophobic interactions were limited to a few π-alkyl interactions with PRO79, ARG76, and ALA90, explaining its reduced binding strength compared to its functionalized counterparts. Table 3 displays the binding affinities of SubPcs with CrtM (PDB ID: 3ACW) from S. aureus. The docking scores for the compounds were: SubPc-1 (−8.592) > SubPc-2 (−4.304) > SubPc-3 (−2.539). According to these results, SubPc-1 demonstrated the highest binding affinity. It formed multiple hydrogen bonds, including conventional hydrogen bonds with HIS18, ARG265, and several pi-donor hydrogen bonds with ASP49 and ARG181. The compound also engaged in numerous hydrophobic interactions, including π-π stacked and T-shaped interactions within itself and with residues like PHE22 and TYR41. The extended π-system of the bisthiophene group appears to contribute significantly to these interactions, explaining its superior binding affinity. SubPc-2 showed a considerably lower binding affinity compared to SubPc1. It formed conventional hydrogen bonds with ARG45, HIS18, and ARG265, and engaged in some hydrophobic interactions, primarily alkyl and π-alkyl, with residues such as LEU164 and ILE51. The presence of sulfur atoms in both SubPc-1 and SubPc-2 allowed for pi-sulfur interactions, which may contribute to their binding, although this effect seems more pronounced in SubPc-1. SubPc-3 exhibited the lowest binding affinity. It formed conventional hydrogen bonds with ARG45/ASP48 and engaged in a π-π T-shaped interaction with HIS18. However, its overall interaction profile was less extensive compared to SubPc-1 and SubPc-2.

**Table 2:**
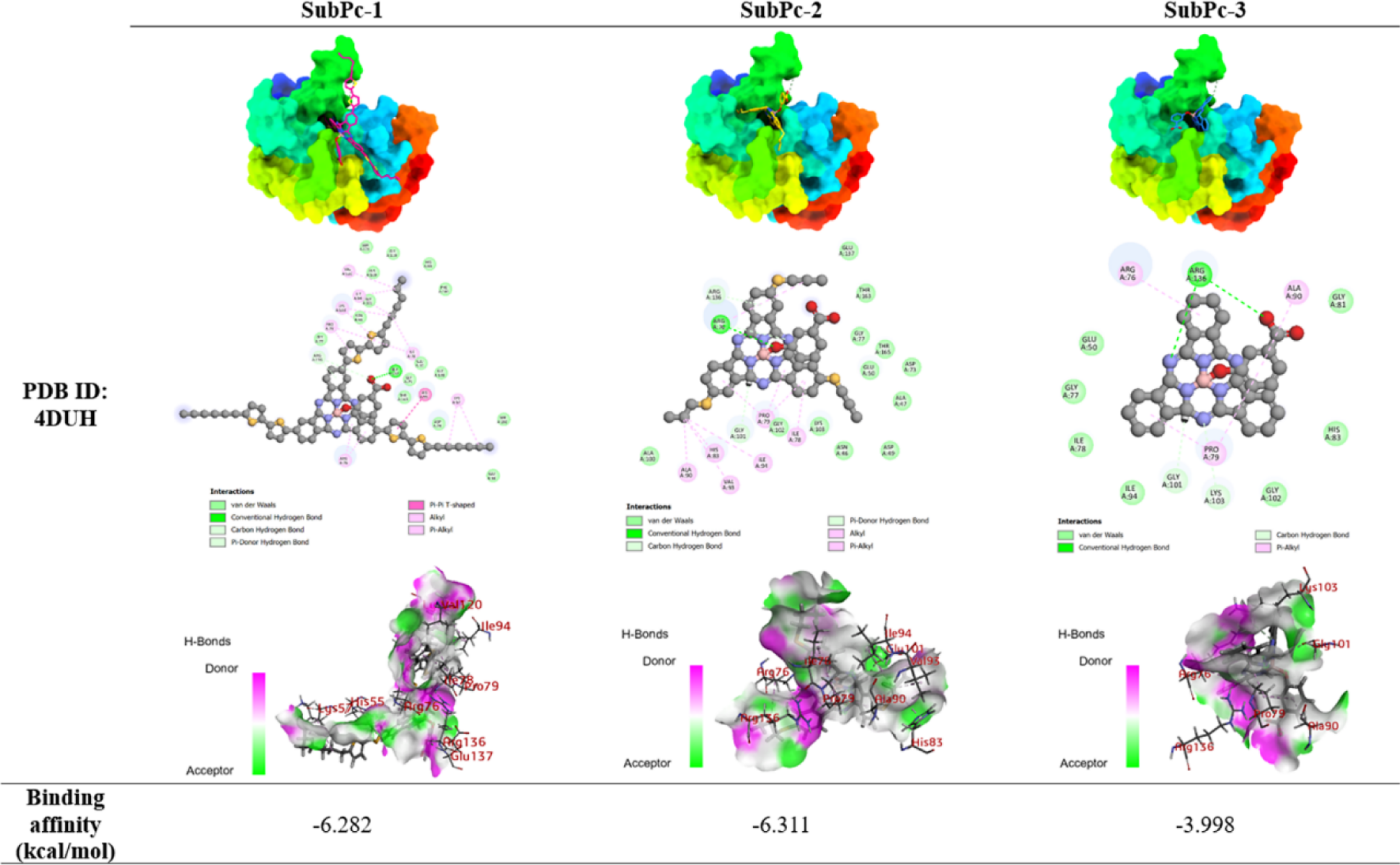
Binding Poses and Residue Interactions of the SubPcs with DNA Gyrase (PDB ID: 4DUH) from E. coli

**Table 3:**
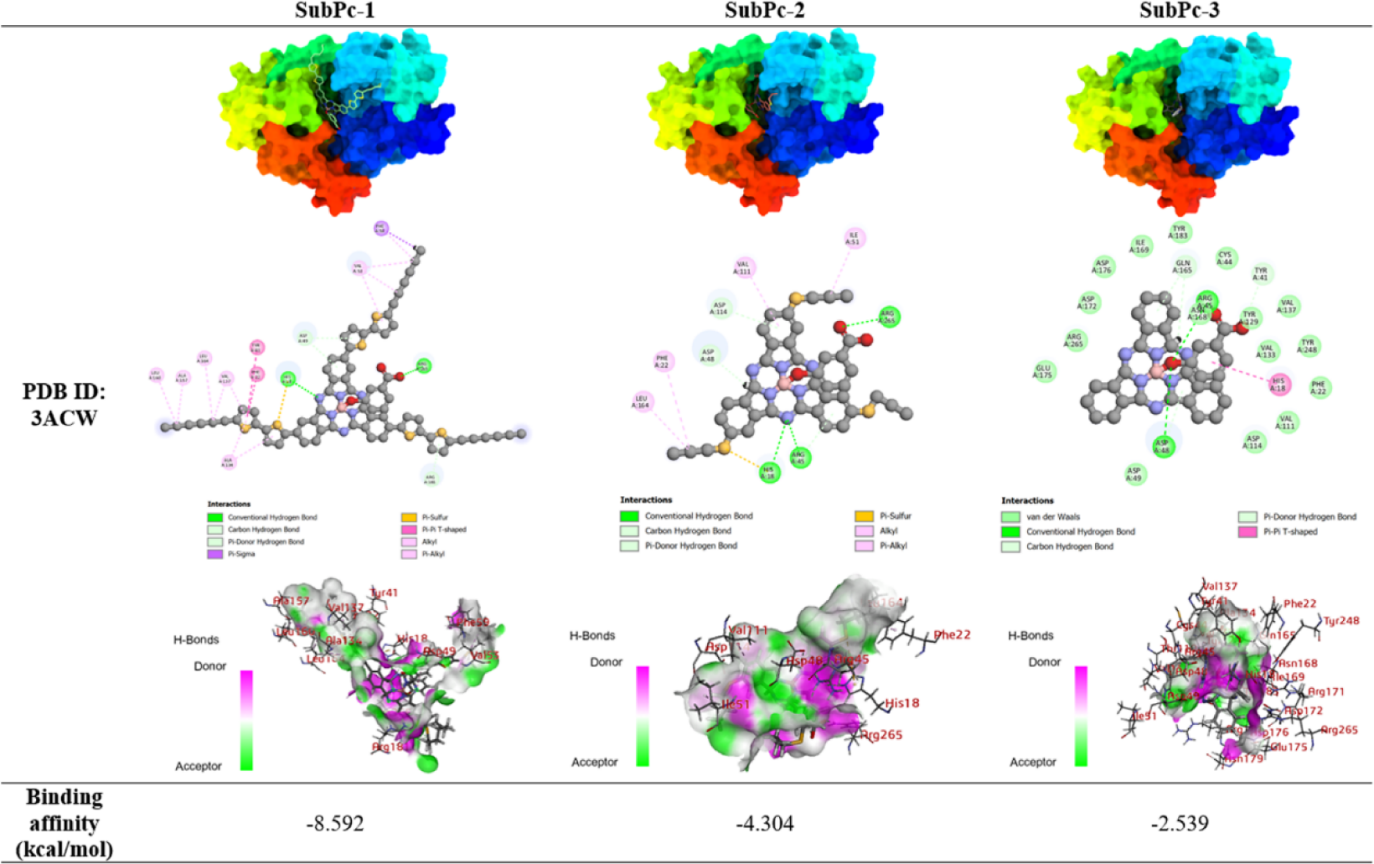
Binding Poses and Residue Interactions of the SubPcs with Dehydrosqualene Synthase (CrtM, PDB ID: 3ACW) from S. aureus

The molecular docking results not only provide a theoretical framework for understanding the interactions of SubPc derivatives with key bacterial proteins but also correlate strongly with the experimental data obtained from antibacterial assays. For instance, the strong binding affinity of SubPc-1 for CrtM aligns with its demonstrated efficacy against S. aureus, as evidenced by a nearly 100% reduction in bacterial viability under LED light. Similarly, the significant interaction of SubPc-2 with PBP2a parallels its observed potency against MRSA biofilm formation, further supporting its potential as a therapeutic agent. Collectively, these findings suggest that the antibacterial mechanisms of SubPc derivatives involve both the generation of reactive oxygen species and the direct inhibition of critical bacterial proteins, reinforcing the importance of integrating computational and experimental methodologies in elucidating drug action. Detailed molecular docking parameters including binding energies, bond distances, involved amino acids, and interaction types for all SubPc derivatives and bacterial target proteins are provided in Table S4.

### 3.8 Molecular Dynamics

In this section, we delve into the comprehensive analysis of the molecular dynamics (MD) simulations performed on the SubPc derivatives in complex with their respective bacterial protein targets. Our investigation focuses on three key parameters that provide crucial insights into the stability, conformational dynamics, and structural integrity of these complexes: Root Mean Square Deviation (RMSD), Solvent Accessible Surface Area (SASA), and Radius of Gyration (Rg). These metrics, widely recognized in the field of computational biophysics, offer complementary perspectives on the behavior of protein-ligand systems over time. RMSD analysis allows us to assess the overall structural stability and conformational changes of the complexes (Table S1). SASA measurements provide information on the extent of protein–ligand interactions and potential changes in solvent exposure (Table S2). Lastly, Rg calculations (Table S2) offer insights into the compactness and global conformational states of the protein-ligand complexes. By integrating these analyses, we aim to elucidate the molecular mechanisms underlying the differential binding affinities and stabilities observed in our docking studies, thereby enhancing our understanding of the SubPc derivatives’ potential as antibacterial agents.

For PBP2a from MRSA (1MWT), the RMSD trajectories reveal distinct stability profiles for each SubPc derivative. The SubPc-1 complex exhibits significant fluctuations (0.4–1.2 nm), indicative of dynamic interactions or induced-fit phenomena. This behavior aligns with studies on PBP2a inhibitors that demonstrate conformational flexibility upon ligand binding.^36^ Conversely, the SubPc-2 complex demonstrates remarkable stability (∼0.5 nm), suggesting a tight, consistent binding mode that corroborates its superior docking score (−7.032). The SubPc-3 complex displays a gradual RMSD increase up to ∼3.5 nm, potentially indicating significant conformational changes or partial unbinding events, consistent with its lower affinity (−5.013). The CrtM protein from S. aureus (3ACW) exhibits distinct interaction patterns with the SubPc derivatives. All complexes show initial equilibration followed by relatively stable RMSD profiles. The SubPc-1 complex stabilizes around 1 nm, indicating a consistent binding mode that aligns with its exceptional docking score (−8.592). This stability echoes findings from recent studies on CrtM inhibitors, where compounds with extended π-systems demonstrated enhanced binding affinities and induced specific protein conformations (10.3389/fmicb.2023.1279082). SubPc-2 and SubPc-3 complexes exhibit lower RMSD values (0.4–0.6 nm), suggesting tighter, though possibly less extensive, interactions. For DNA gyrase from E. coli (4DUH), all three SubPc derivatives display similar RMSD patterns (0.6–1.0 nm), indicating comparable binding modes. The slightly higher RMSD values observed for SubPc-1 and SubPc-2 complexes compared to SubPc-3 may suggest more extensive interactions or induced-fit effects. This observation aligns with recent studies on gyrase inhibitors that demonstrate the importance of ligand-induced conformational changes in achieving potent inhibition.^37^ Across all systems, the protein RMSD closely mirrors the complex RMSD, while ligands maintain low RMSD values. These MD simulations provide a more nuanced view of the interactions, highlighting the complex interplay between ligand binding and protein dynamics. The results emphasize that stable complexes can result from both tight, localized interactions (as seen with SubPc-2 in PBP2a) and more extensive, dynamic binding modes (exemplified by SubPc-1 in CrtM). This dynamic perspective aligns with modern concepts in drug design, where ligand-induced protein flexibility is increasingly recognized as a critical factor in achieving specificity and potency (Table S1).

The Solvent Accessible Surface Area (SASA) trajectories provide crucial insights into the conformational dynamics and protein-ligand interactions of the SubPc derivatives (Table S2).

In the case of PBP2a from MRSA (1MWT), all three SubPc complexes exhibit relatively stable SASA profiles, fluctuating around 620–630 nm^2^. SubPc-1 shows slightly higher SASA values and more pronounced fluctuations compared to SubPc-2 and SubPc-3. This observation suggests that SubPc-1 may induce a more open conformation of PBP2a, potentially exposing additional surface area to the solvent. The stability of SASA for SubPc-2 and SubPc-3 complexes indicates consistent protein-ligand interactions throughout the simulation, corroborating the RMSD data that showed SubPc-2’s remarkable stability.

The CrtM protein from S. aureus (3ACW) displays distinct SASA patterns for each SubPc derivative. The SubPc-1 complex exhibits a gradual increase in SASA over time, reaching values around 160 nm^2^ by the end of the simulation. This trend suggests that SubPc-1 may induce conformational changes in CrtM that lead to increased solvent exposure, possibly reflecting the formation of a more extensive binding interface. In contrast, SubPc-2 and SubPc-3 complexes maintain relatively stable SASA values around 150–155 nm^2^, indicating more conserved protein conformations throughout the simulation.

For DNA gyrase from E. coli (4DUH), all three SubPc complexes demonstrate similar SASA profiles, fluctuating between 105–115 nm^2^. The consistency in SASA values across the three complexes suggests that the SubPc derivatives interact with DNA gyrase in a manner that does not significantly alter the protein’s overall solvent exposure. However, subtle differences are observed, with SubPc-1 showing slightly higher and more variable SASA values compared to SubPc-2 and SubPc-3. This observation aligns with the RMSD data, indicating that SubPc-1 may engage in more dynamic interactions with the protein. The SASA analysis complements the RMSD data by providing insights into the surface characteristics of the protein-ligand complexes.

The relatively stable SASA profiles observed across most systems indicate that the SubPc derivatives generally maintain consistent interactions with their target proteins without inducing large-scale conformational changes that would significantly alter solvent accessibility. However, the subtle differences observed, particularly for SubPc-1 in the CrtM complex, highlight the compound’s potential to induce specific conformational changes that may contribute to its enhanced binding affinity.

The Radius of Gyration (Rg) trajectories provide valuable insights into the overall compactness and global conformational changes of the protein-ligand complexes (Table S3). The Rg profiles of each SubPc derivative exhibit unique behaviors for PBP2a from MRSA (1MWT). SubPc-1 exhibits a gradual increase in Rg from approximately 4.05 nm to 4.15 nm over the course of the simulation, suggesting a slight expansion of the protein structure. This observation aligns with the higher SASA values noted earlier, indicating that SubPc-1 may induce a more open conformation of PBP2a. In contrast, SubPc-2 and SubPc-3 show more stable Rg values, fluctuating around 4.05 and 4.00 nm, respectively. The lower and more consistent Rg values for SubPc-2 corroborate its superior stability observed in the RMSD analysis, implying a tighter, more compact protein-ligand complex.

The CrtM protein from S. aureus (3ACW) displays relatively consistent Rg profiles across all three SubPc derivatives, with values oscillating around 2.05 nm. This stability in Rg suggests that the overall protein compactness is maintained regardless of the bound SubPc variant. However, subtle differences are observable. SubPc-1 shows slightly more variability in Rg, particularly toward the end of the simulation, which may correspond to the increased SASA values noted earlier. This could indicate that while the overall protein compactness is maintained, SubPc-1 induces local conformational changes that affect surface exposure without significantly altering the global protein structure.

For DNA gyrase from E. coli (4DUH), all three SubPc complexes demonstrate remarkably stable Rg profiles, with values consistently around 1.65 nm. This uniformity across the different SubPc derivatives suggests that they interact with DNA gyrase in a manner that preserves the protein’s global conformation. The stability in Rg aligns with the consistent SASA profiles observed earlier, reinforcing the notion that the SubPc compounds do not induce large-scale conformational changes in DNA gyrase.

These Rg analyses provide crucial information about the global structural stability of the protein-ligand complexes. The observed patterns indicate that while local conformational changes may occur (as evidenced by RMSD and SASA data), the overall compactness of the proteins is largely maintained. This stability is particularly noteworthy for the DNA gyrase complexes and suggests that the SubPc derivatives may achieve their inhibitory effects through localized interactions rather than by inducing large-scale structural reorganizations.

## 4 Conclusion

The research demonstrates that nontoxic SubPc-linked TiO_2_ materials exhibit potent photocatalytic antibacterial and antibiofilm effects under LED light, with SubPc-1–2/TiO_2_ showing nearly 100% efficacy against S. aureus, followed by SubPc-3/TiO_2_. These materials demonstrated significantly higher antibacterial efficacy under LED light compared to dark conditions, not only against S. aureus but also against E. coli and MRSA. Molecular docking and dynamics analyses revealed varying affinities for key bacterial proteins: SubPc-1 strongly binds to CrtM in S. aureus, while SubPc-2 shows high affinity for PBP2a in MRSA. The SubPc derivatives operate through a dual mechanism: generating reactive oxygen species through photocatalysis and directly targeting bacterial proteins. This comprehensive approach, combining experimental photocatalytic antibacterial testing with computational methods, provides a thorough understanding of both ROS-driven antibacterial mechanisms and molecular interactions between SubPc derivatives and bacterial proteins. These findings collectively establish these materials as promising candidates for treating infectious diseases and antibiotic-resistant bacteria like MRSA, while opening possibilities for developing next-generation photosensitizers for various photocatalytic applications. The use of nanomaterials in alternative antibiotic strategies is gaining prominence. In this study, nontoxic SubPc-linked TiO_2_ materials showed strong photocatalytic antibacterial and antibiofilm activity under LED light, with SubPc-3/TiO_2_ achieving nearly 100% inhibition of S. aureus. SubPc-2 and SubPc-1 followed in efficacy. These materials were also effective against E. coli and MRSA, with significantly higher activity under light than in the dark. The highest GSH consumption (82%) observed with SubPc-3/TiO_2_ indicates strong ROS involvement. Fluorescence microscopy and SEM confirmed antibacterial effects and morphological changes. The mechanism was explored via electronic band analysis and ROS formation. Molecular docking revealed strong interactions between SubPc derivatives and bacterial targets: SubPc-1 with CrtM from S. aureus, and SubPc-2 with PBP2a from MRSA. These findings were supported by 100 ns molecular dynamics simulations, RMSD, SASA, and Rg analyses, indicating stable protein-ligand complexes. Combining experimental and computational data, the study shows that SubPc derivatives act via dual mechanisms: ROS generation and bacterial protein targeting. This dual action makes them promising candidates against antibiotic-resistant bacteria like MRSA. Overall, the study offers a solid foundation for developing advanced photosensitizers for treating infectious diseases and enhancing photocatalytic applications.

## Supplementary Material



## Notes

SEM images of SubPc/TiO_2_ structures (Figure S1); fluorescent microscopy images of bacterial strains under dark and LED conditions (Figure S2); RMSD trajectories (Table S1); SASA trajectories (Table S2); radius of gyration data (Table S3); molecular docking parameters including binding energies, interaction types, bond distances, and amino acids involved (Table S4) (PDF)
