# Supplementary material for: Photocatalytic Antibacterial and Antibiofilm Activities of Subphthalocyanine/TiO2 Derivates: Integrating Experimental Findings with Molecular Docking and Dynamics
Source: ACS Omega. 2025 May 28;10(22):23182–95. doi: 10.1021/acsomega.5c01465 (PMC12163689; doi:10.1021/acsomega.5c01465)
Supplement: Supplementary file 1 [file ao5c01465_si_001.pdf]

SUPPORTING INFORMATION

Photocatalytic Antibacterial and Antibiofilm Activities of Subphthalocyanine/TiO_2 Derivates: Integrating Experimental Findings with Molecular Docking and Dynamics

Buket Guntay[a], Tugce Ozcan[b], Şifa Doğan[c], İlknur Aksoy Çekceoğlu[b], Gülbin Kurtay[d*], Emre Aslan[a], Mine Ince[c*], Imren Hatay Patir[b*]

[a] Department of Biochemistry, Selcuk University, 42130, Konya, Turkey

[b] Department of Biotechnology, Selcuk University, 42250, Konya, Turkey

[c] Department of Natural and Mathematical Science, Tarsus University, 33400, Mersin, Turkey

[d] Department of Chemistry, Hacettepe University, 06800, Ankara, Turkey

Corresponding author-1: Gülbin KURTAY (gulbinkurtay@hacettepe.edu.tr)

Corresponding author-2: Mine Ince (mine.ince@tarsus.edu.tr)

Corresponding author-3: Imren Hatay Patir (imrenhatay@gmail.com)

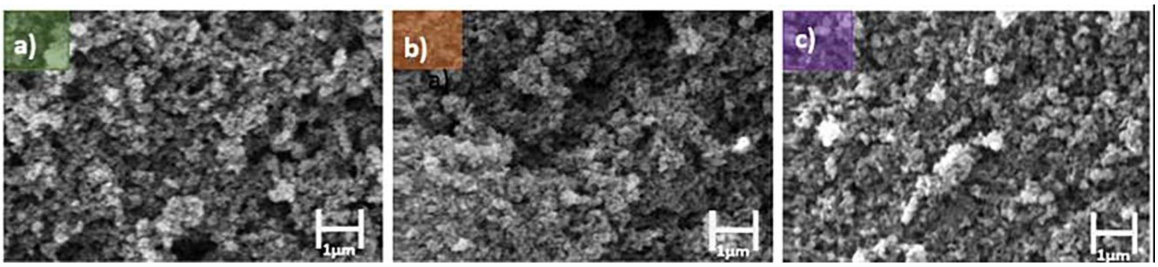

Figure S1. SEM images of SubPc/TiO_2 structures (a: SubPc1/TiO_2, b: SubPc2/TiO2, c: SubPc3/TiO_2)

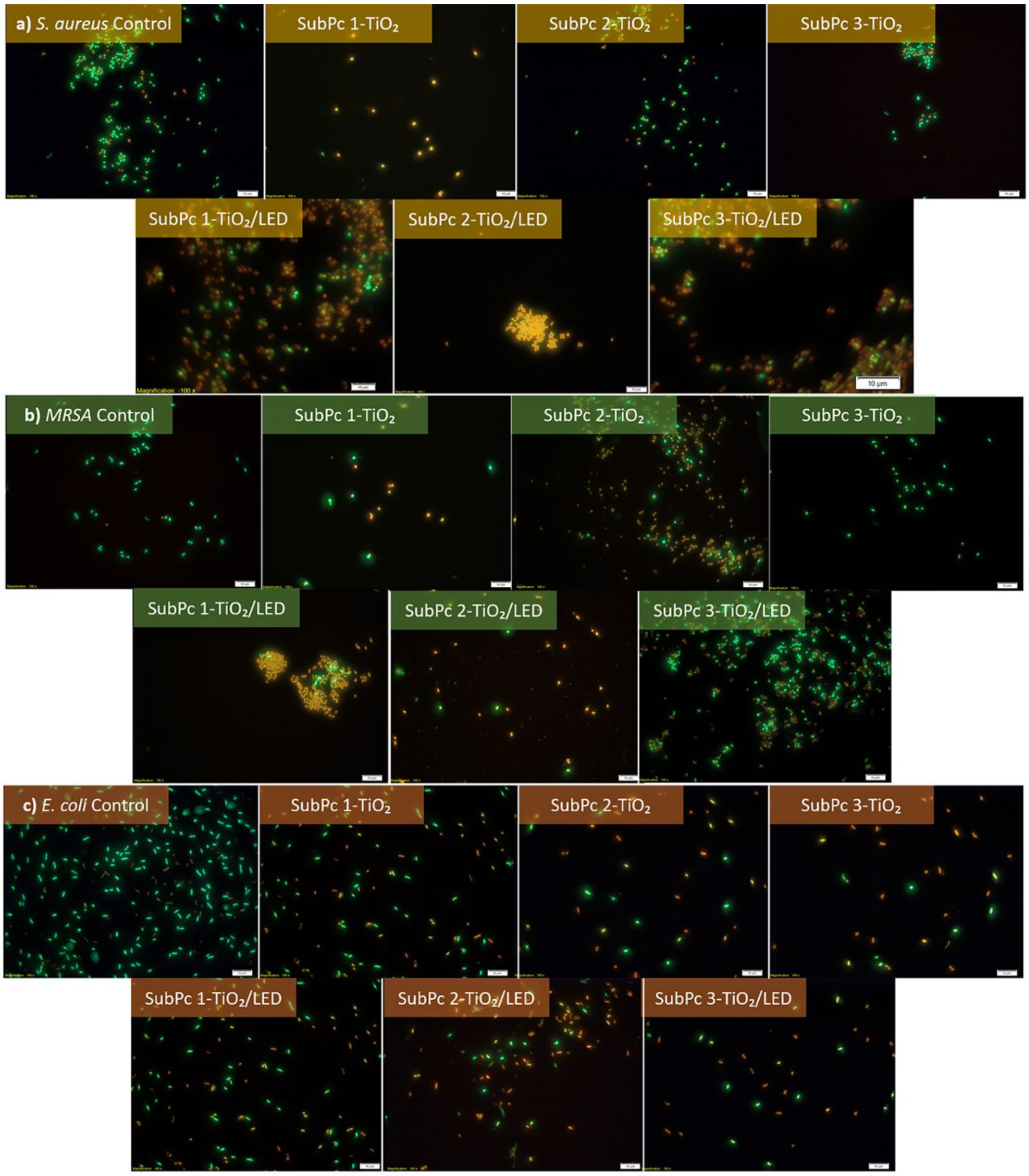

Figure S2. Fluorescent images of *S. aureus* **(a)**, MRSA **(b)**, and *E. coli* **(c)** strains treated with SubPcs/TiO_2 in the presence/absence of LED light illumination

Table S1. RMSD trajectory plots of SubPcs with target proteins (1MWT, 3ACW and 4DUH)

PDB ID	SubPc-1	SubPc-2	SubPc-3
**1MWT**	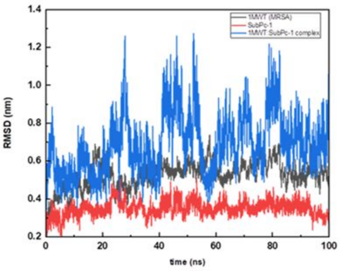	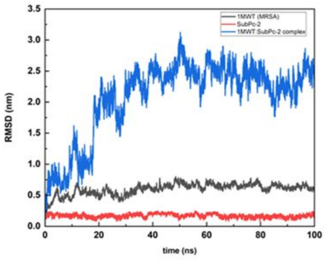	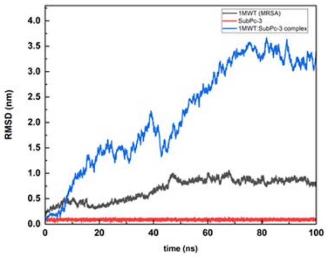
**3ACW**	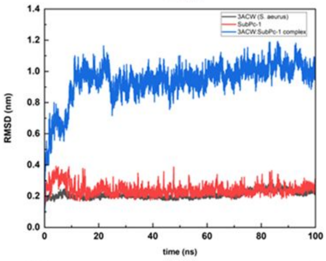	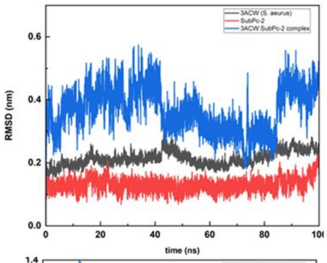	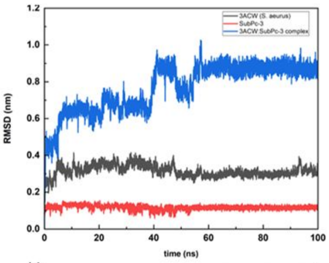
**4DUH**	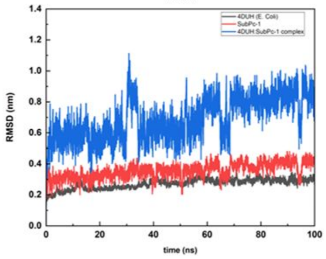	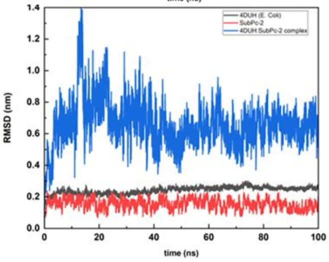	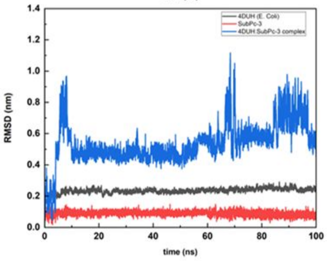

Table S2. SASA trajectories of SubPcs with target proteins (1MWT, 3ACW and 4DUH)

<table>
<tr><th>PDB ID</th><th>SubPc-1</th><th>SubPc-2</th><th>SubPc-3</th></tr>
<tr><td>1MWT</td><td>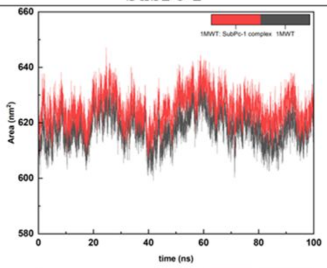
</td><td>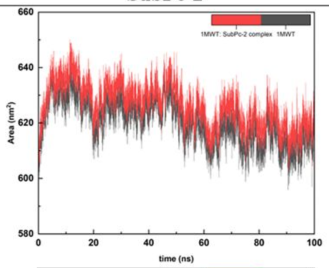
</td><td>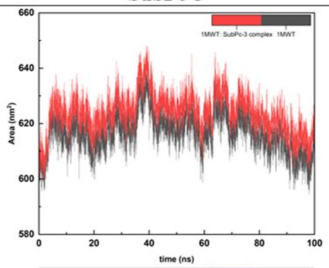
</td></tr>
<tr><td>3ACW</td><td>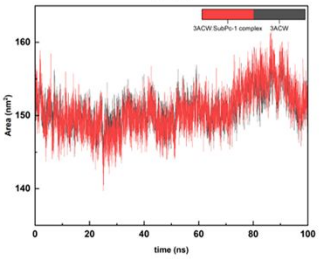
</td><td>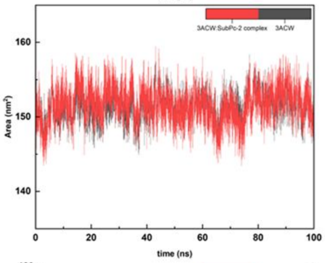
</td><td>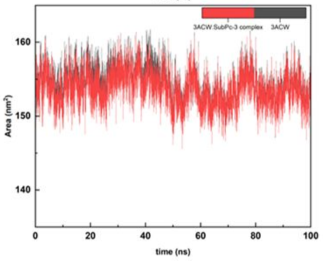
</td></tr>
<tr><td>4DUH</td><td>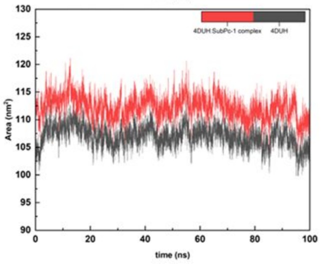
</td><td>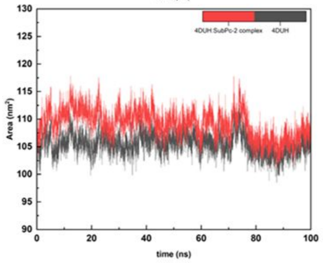
</td><td>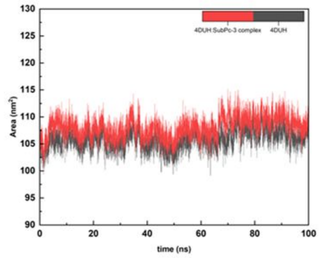
</td></tr>
</table>

Table S3. Radius of gyration graphs of of SubPcs with target proteins (1MWT, 3ACW and 4DUH)

PDB ID	SubPc-1	SubPc-2	SubPc-3
**1MWT**	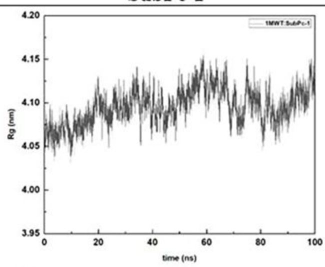	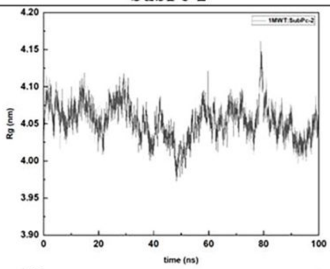	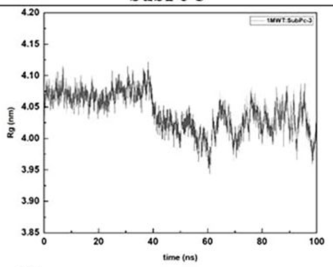
**3ACW**	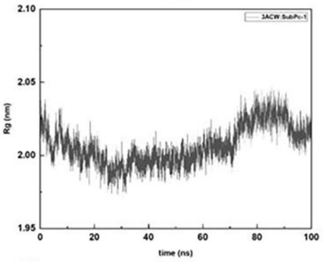	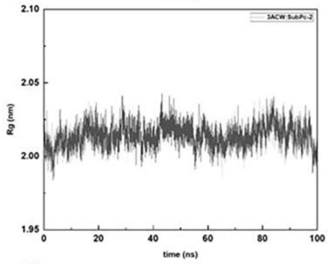	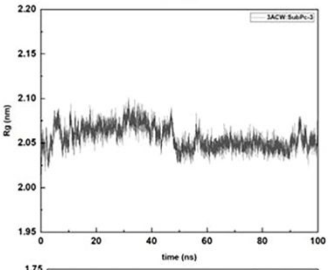
**4DUH**	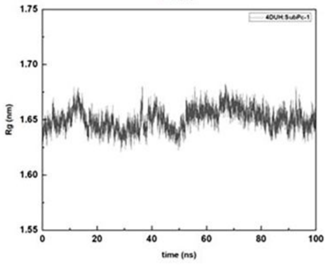	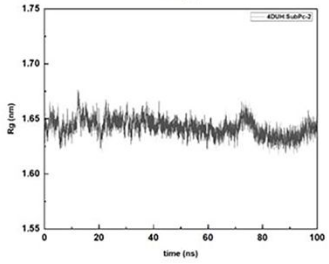	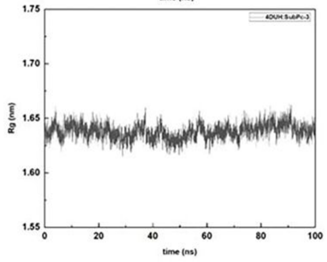

Table S4. Molecular docking parameters including binding energies, interaction types, bond distances, amino acids involved

Compound	Target Protein (PDB ID)	Binding Energy (kcal/mol)	Interaction Type	Bond Distance (Å)	Amino Acids Involved
SubPc-1	PBP2a (1MWT)	-5.801	Conventional Hydrogen Bond	1.70–2.89	LYS430, GLY640
			Pi-Donor Hydrogen Bond	3.67–4.08	GLU602
			Pi-Pi Stacked	3.75–5.46	TYR446
			Amide-Pi Stacked	4	ASP638, LYS639
			Alkyl/Pi-Alkyl	4.98–5.01	ILE512, TYR441
SubPc-2		-7.032	Pi-Pi T-shaped	4.65–4.94	TYR446, HIS583
			Alkyl	3.92	VAL448
SubPc-3		-5.013	Conventional Hydrogen Bond	1.85–2.00	LYS430, ASN464
			Carbon Hydrogen Bond	2.31–2.91	LYS430, TYR446, THR444
			Pi-Sigma	2.75	TYR446
			Pi-Sulfur	5.22–5.23	MET641
			Pi-Pi Stacked	5.25	TYR446
			Pi-Alkyl	5.22	LYS430
SubPc-1	DNA gyrase (4DUH)	-6.282	Conventional Hydrogen Bond	1.73–2.92	ARG76, ARG136
			Carbon Hydrogen Bond	2.58	ARG76
			Pi-Pi Stacked	5.37	Ligand–Ligand
			Alkyl	4.37–5.42	ALA47, VAL120, ILE78, VAL43, VAL71
			Pi-Alkyl	3.93–5.42	PRO79, PRO84, ILE78, ILE82
SubPc-2		-6.311	Conventional Hydrogen Bond	1.91	ARG136
			Water-mediated Hydrogen Bond	1.59	HOH702
			Carbon Hydrogen Bond	2.71	ARG76
			Alkyl	3.97–5.04	LYS103, ILE78
			Pi-Alkyl	4.23–5.43	PRO79, LYS103
SubPc-3		-3.998	Conventional Hydrogen Bond	1.75–1.80	ARG136
			Water-mediated Hydrogen Bond	2.03	HOH702
			Carbon Hydrogen Bond	2.33	ARG76
			Pi-Pi Stacked	3.89	HIS83
			Amide-Pi Stacked	4.29	GLY101, GLY102
			Pi-Alkyl	4.11–5.47	ALA90, VAL93, ILE94, ALA100, PRO79
SubPc-1	CrtM (3ACW)	-8.592	Conventional Hydrogen Bond	1.79–2.97	HIS18, ARG265
			Carbon Hydrogen Bond	3.09	HIS18
			Pi-Donor Hydrogen Bond	2.89–4.09	ASP49, ARG181
			Pi-Pi Stacked/T-shaped	3.95–5.41	PHE22, TYR41
			Alkyl/Pi-Alkyl	4.03–5.28	VAL53, VAL137, ALA157, LEU160, LEU164, ALA134, PHE59
SubPc-2		-4.304	Conventional Hydrogen Bond	1.86–2.97	HIS18, ARG45, ARG265
			Carbon Hydrogen Bond	2.75	ASP48
			Pi-Donor Hydrogen Bond	3.07–4.16	ARG45, ASP114
			Alkyl/Pi-Alkyl	4.20–5.10	LEU164, ILE51, PHE22, VAL111
SubPc-3		-2.539	Conventional Hydrogen Bond	1.94–3.10	ARG45, ASP48
			Carbon Hydrogen Bond	2.61–2.77	ARG45, GLN165
			Pi-Donor Hydrogen Bond	2.69–4.11	ASP48, GLN165, TYR41
			Pi-Pi T-shaped	4.51	HIS18
			Pi-Alkyl	4.11–5.47	ALA90, VAL93, ILE94, ALA100, PRO79
			Pi-Sulfur	4.80–5.58	HIS18
